# Long-term outcomes of tracheal stents removal under fluoroscopy guidance: comparison of tracheal fistulas and tracheal stenosis

**DOI:** 10.1186/s12890-020-01349-7

**Published:** 2021-01-07

**Authors:** Yonghua Bi, Jindong Li, Liangliang Bai, Xinwei Han, Jianzhuang Ren

**Affiliations:** 1 Department of Interventional Radiology, The First Affiliated Hospital of Zhengzhou University, No.1, East Jian She Road, Zhengzhou, 450052 China; 2 Department of Thoracic Surgery, The First Affiliated Hospital of Zhengzhou University, Zhengzhou, China

**Keywords:** Respiratory tract fistula, Tracheal stenosis, Stents removal, Fluoroscopy, Postoperative complications

## Abstract

**Background:**

Endoscopic removal is the most common method for removal of tracheal stents. Few studies have reported the technique of fluoroscopy-guided stent removal for tracheal fistula and tracheal stenosis. We aimed to study the safety and efficacy of fluoroscopy-guided stent removal as well as the optimal duration for stent usage.

**Methods:**

We conducted a retrospective analysis of 152 patients who underwent fluoroscopy-guided stent removal from January 2011 to June 2017. Reasons for stent implantation were tracheal fistula in 85 patients (TF group), and tracheal stenosis in 67 patients (TS group). All patients underwent tracheal CT scans before stent removal and during follow up. The technical success rate, complications, and survival rate were compared between the two groups.

**Results:**

The technical success rate of stent removal was 98.9 and 97.4%, respectively for the TF and TS group. Removal was routine for half of patients, and in the remainder, excessive granulation tissue was the common indications for stent removal, which was found after stenting at 142.1 ± 25.9 days in the TF group, and at 89.9 ± 15.0 day in the TS group. The total incidence of complications was 21.1 and 22.4%, respectively, for the TF and TS groups. Perioperative death occurred in one patient in the TF group, and two patients in the TS group. Recurrence of fistula or stenosis requiring re-stenting was the most comment complication in both groups. The 0.5-, 3-, 6-year survival rates were 90.3, 59.6, and 36.1% for TF group, and 80.4, 75.7, 75.7% for TS group.

**Conclusions:**

Fluoroscopic removal of tracheal stents is safe and effective for both tracheal fistula and tracheal stenosis, with no significant difference in outcomes. Clinicians should pay attention to the risk of hemoptysis for patients with malignant tumors and a combination with endoscopic hemostasis may help improve its safety.

**Supplementary Information:**

The online version contains supplementary material available at 10.1186/s12890-020-01349-7.

## Background

Tracheal fistula and/or tracheal stenosis are severe complications after open thoracic surgery, endotracheal intubation injury, endobronchial tuberculosis, and thoracic trauma [1]. These complications often show high rates of mortality and disability rates. Unfortunately, traditional conservative treatment shows a poor curative effect. Although tracheal resection with primary anastomosis is the standard of care for the treatment of tracheal stenosis, tracheal resection is not suitable for long segmental stenosis. Additionally, some patients may not be willing to undergo open surgery because they are too weak or have significant comorbidities [2]. Since self-expanding metal stents were first used to treat bronchial obstruction in 1989 [3], the efficacy and safety of tracheal stents has been proven for the treatment of tracheal fistula [4–7] or tracheal stenosis [8–13]. However, tracheal stent placement may be accompanied by a series of complications, such as restenosis, migration, fracture, or mucous plugging [14], which make stent removal necessary. Endoscopic removal is the most common method reported for removal of tracheal stents [15–20]. Few studies have reported the technique of tracheal stent removal under fluoroscopic guidance [12, 21, 22]. Is fluoroscopic removal of tracheal stents safe and effective? How long after implantation should the tracheal stent be removed? Topics of ongoing investigation include the safety and efficacy of fluoroscopy-guided stent removal as well as the optimal duration for stent usage. We compared the outcomes and complications associated with fluoroscopy-guided removal of tracheal stents implanted for tracheal fistula and tracheal stenosis.

## Methods

### Patients

This retrospective study was approved by the Ethics Committee and Medical Records Management Section of our University, and informed consent was waived. We retrospectively reviewed the records of a total of 152 patients who underwent fluoroscopy-guided stent removal from January 2011 to June 2017 in our department. Of these, 85 patients underwent removal of stents implanted for tracheal fistula (TF group), and 67 patients underwent removal of stents implanted for tracheal stenosis (TS group). More than 50% of patients underwent routine removal in both groups to avoid long-term complications even if they show no obvious symptoms or signs.

### Tracheal stent

**Fig. 1:**
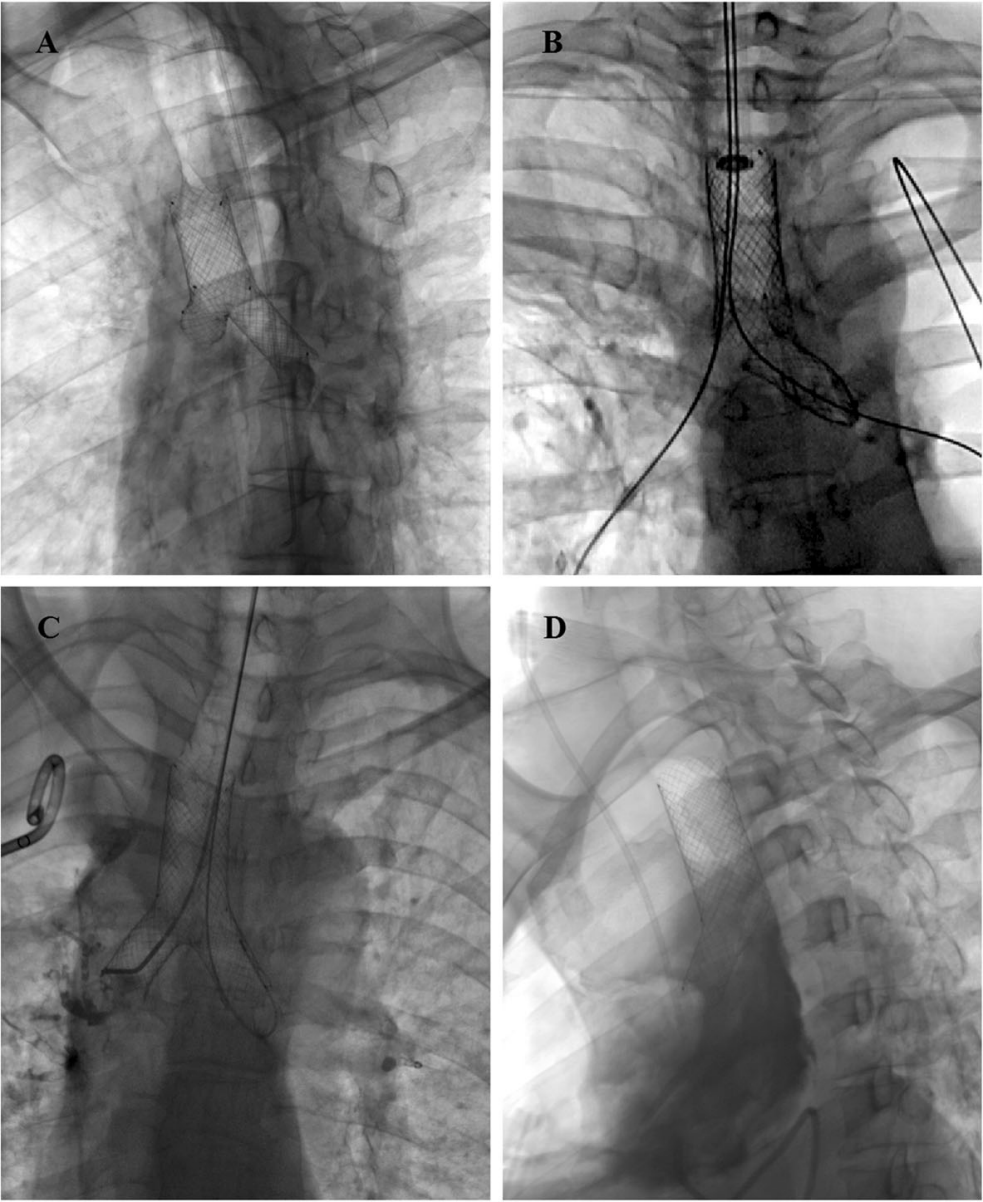
Types of individualized tracheal stents under fluoroscope. **a** Y-shaped single-plugged tracheal stent, **b** plugged bullet-shaped tracheal stent, **c** Y-type tracheal stent, **d** L-type tracheal stent

Tracheal stents were designed and manufactured according to individual tracheal shape and size measured by CT examination (Nanjing Micro-Tech Medical Company, Nanjing, China), and woven with a temperature-memory nickel–titanium alloy wire. All tracheal stents for fistula were fully covered in TF group, and 70 covered stents and 6 bare stents were used in TS group (Fig. 1). Ninety tracheal stents were implanted in TF group, and large Y-shaped single-plugged tracheal stent was the most common type (32.2%). In TS group, 76 stents were implanted and the most common type was straight tracheal stent (73.7%).

### Technical preparation before and after tracheal stent removal

Chest CT and bronchoscopy were performed on the day before stent removal. Electrocardiograms, heart rate and blood pressure were routinely monitored during the perioperative period. Oxygen was given by nasal catheter and a sputum aspirator was prepared. Diazepam, 10 mg, and anisodamine (654–2) was injected intramuscularly to calm the patient and to reduce tracheal secretions before stent removal. Dexamethasone, 10 mg, was injected to relieve dyspnea or to improve tolerability if necessary. The sputum aspiration tube was then advanced into the tracheal for sputum drainage and to maintain tracheal patency. All patients were monitored by electrocardiogram, heart rate and blood pressure for at least 8 h after stent removal.

### Technical details of tracheal stent removal

**Fig. 2:**
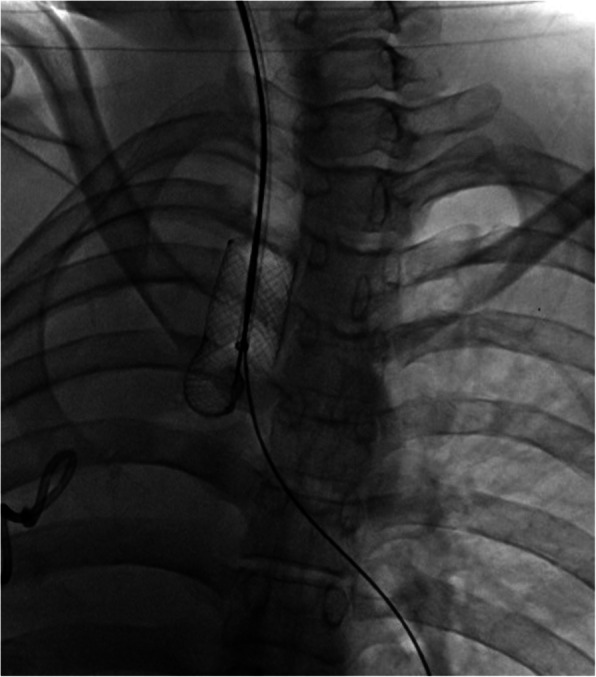
Technique of tracheal stent removal. A retrieval hook was inserted through a sheath and the tip of hook was placed next to the proximal end of the stent. The stent then was carefully dissected from the tracheal wall

All interventional procedures were performed under fluoroscopy and by two to three clinically experienced experts with at least 10 years of experience in interventional treatment for airway disease. A 5F catheter and a 0.035 in. hydrophilic guide wire (Cook Corporation, Bloomington, IN, USA) were inserted into the main bronchus under topical anesthesia with lidocaine. A 10-12F long sheath was inserted below the tracheal stent via a 0.035 in. stiff hydrophilic guide wire. A removal hook was introduced slowly along the sheath with its tip placed next to the distal end of stent. The whole procedure of stent removal was carried out under continuous fluoroscopy to closely monitor and to prevent complications such as stent shedding. The tracheal stent was hooked firmly and then withdrawn carefully (Fig. 2, Video 1, Video 2). Radiography was performed again to show whether there was an overflow of contrast material after stent removal. For patients with severe stenosis within the stent due to granulation tissue, preprocedural bronchoscopy was performed and high-frequency electrosurgical excision, cryotherapy or an argon knife were used to decrease the amount of granulation tissue. Thereafter, stent removal was performed.

### Complications of stent removal and follow up

All complications of stent removal were recored and analyzed. Patients were followed up after stent removal, and chest CT scans were performed. Telephone follow-up was used for patients who did not come to the hospital for reexamination.

### Statistical analysis

Data were expressed as mean ± standard deviation, and analyzed by student t test and ANOVA. Qualitative data were expressed in percentage, and analyzed by Fisher’s exact test. Patency rate was compared by Log-rank (Mantel-Cox) Test (GraphPad Software, Inc., USA). Statistical significance was considered when *p* < 0.05.

## Results

### Patient characteristics

Ninety covered tracheal stents were inserted in the TF group and 76 tracheal stents were implanted in the TS group. Tracheal fistulas were caused by malignant tumor invasion or surgical removal in 81.2% patients in the TF group. There were 47 cases of lung cancer, 2 cases of thyroid cancer and 20 cases of esophageal squamous cell carcinoma. Among lung cancer, 4 cases of lung adenocarcinoma, 1 case of lung adenosquamous carcinoma, the rest are lung squamous cell carcinoma. Tracheal stenosis was caused mainly by benign primary disease requiring tracheotomy or tracheal cannula in 82.1% patients in the TS group. There were 5 cases of lung squamous cell carcinoma, 3 cases of thyroid cancer and 4 cases of esophageal squamous cell carcinoma. The success rate of stent removal was 98.9% (89/90) in the TF group. Only one patient underwent bronchoscopic removal for retained stent pieces after failure of fluoroscopic removal. In the TS group, the success rate of stent removal was 97.4% (74 of 76 stents) and only 2 stents retained. The mean procedure time for stent implantation or removal was similar between two groups. Tumor invasion or tumor operation was the main causes for tracheal fistula, and tracheotomy or trachea cannula was the main causes for tracheal stenosis (*p* < 0.0001, Table 1).

**Table 1:** Patient characteristics

Characteristics	TF group	TS group	*p*
Patients, N	85	67	
Mean age (range), years.	54.9 ± 1.3 (15–81)	51.1 ± 2.2 (12–85)	0.1306
Male/female gender, N	72/13	39/28	0.0004
Malignancy/benign primary disease, N	69/16	12/55	< 0.0001
Procedure time of implantation	29.1 ± 1.6 (10–53)	26.8 ± 1.9 (9–60)	0.3598
Procedure time of stent removal	19.0 ± 1.3	23.3 ± 2.0	0.0715
Causes of disease, N (%)			
Tracheotomy/Trachea cannula	0 (0%)	32 (47.8%)	< 0.0001
Tumor invasion/Tumor operation	69 (81.2%)	12 (17.9%)	< 0.0001
External pressure	0 (0%)	7 (10.4%)	0.0027
Surgery for inflammatory disease	9 (10.6%)	6 (9.0%)	0.7908
Tracheal stent implantation	0 (0%)	5 (7.5%)	0.0153
Tracheal mechanical injury or trauma	3 (3.5%)	3 (4.5%)	1.0000
Others	4 (4.7%)	2 (3.0%)	0.6947

*N* number

### Indications for stent removal and interval

Forty-seven stents implanted for tracheal fistula and 40 stents implanted for tracheal stenosis were routinely removed. Stent migration and stent intolerance were the main indications for early stent removal in both groups. Excessive granulation tissue increased as the time interval after implantation increased, with a mean interval of 142.1 ± 25.9 days for tracheal fistula and 89.9 ± 15.0 day for tracheal stenosis, and was the most common indications for later stent removal. Interestingly, tracheal stents were routinely removed after 107.8 ± 9.8 days and 85.4 ± 6.7 days, respectively, in the TF group and the TS group (Table 2). These two time points are consistent, suggesting that the stent should be removed about 3 months after implantation to reduce the incidence of restenosis. Six bare stents were implanted on an emergency basis in the TS group for severe tracheal stenosis. Three of these stents were removed and replaced by covered stents, with a mean duration of 8.7 ± 3.2 days, and one stent was replaced by a tracheal T-tube.

**Table 2:** Indications of stent removal and interval

	TF group	TS group	*p*
Indications of removal, N (%)			
Routine removal	47 (52.2%)	40 (52.6%)	1.0000
Excessive granulation tissue	11 (12.2%)	15 (19.7%)	0.2041
Stent migration	12 (13.3%)	5 (6.6%)	0.2011
Intolerance of stenting	8 (8.9%)	6 (7.9%)	1.0000
Inadequate expansion and deformation	0 (0.0%)	5 (6.6%)	0.0187
Strut fracture	4 (4.4%)	1 (1.3%)	0.3764
Recurrence of fistula	8 (8.9%)	–	–
Replacement of bare stent	–	4 (5.3%)	–
Interval between placement and removal, days			
Routine removal	107.8 ± 9.8	85.4 ± 6.7	0.0703
Excessive granulation tissue	142.1 ± 25.9	89.9 ± 15.0	0.0744
Stent migration	20.2 ± 8.1	27.0 ± 19.2	0.7003
Intolerance of stenting	18.9 ± 10.5	8.3 ± 4.3	0.4256
Inadequate expansion and deformation	–	2.0 ± 2.0	–
Strut fracture	97.0 ± 14.8	105	–
Recurrence of fistula	60.6 ± 36.7	–	–
Replacement of bare stent	–	8.7 ± 3.2	–
Total (range)	89.5 ± 8.3 (0–435)	68.1 ± 6.1 (0–221)	0.0439

### Complications

The technical success rate of stent removal was 98.9 and 97.4%, respectively, for TF and TS groups. In the TF group, 75 stents were removed in one piece. Stent fracture and retained stent pieces were found in 14 patients and 1 stent. In the TS group, 74 tracheal stents were successfully removed, including 71 in one piece and 5 with strut fracture. Two of these stents were retained and the other three retained stent pieces were successfully removed by endoscopy (Table 3).

**Table 3:** Complications of stent removal

Complication	TF group	TS group	*p*
Recurrence requires stenting	13 (14.4%)	9 (11.8%)	0.6538
Y-type tracheal stent	6 (6.7%)	2 (2.6%)	0.2914
Straight tracheal stent	3 (3.3%)	7 (9.2%)	0.1886
L-type tracheal stent	3 (3.3%)	0 (0.0%)	0.2508
plugged bullet-shaped stent	1 (1.1%)	0 (0.0%)	1.0000
Strut fracture and residue	1 (1.1%)	5 (6.6%)	0.0944
Mucosal tear with massive bleeding	2 (2.2%)	1 (1.3%)	1.0000
Reobstruction requiring stenting	1 (1.1%)	0 (0.0%)	1.0000
Dyspne need for mechanical ventilation	2 (2.2%)	2 (2.6%)	1.0000
Total complications	19 (21.1%)	17 (22.4%)	0.8525

*N* number

Recurrence of fistula or stenosis requiring stenting was the most comment complication in both groups. Two patients showed severe dyspnea in each group and underwent endotracheal intubation and mechanical ventilation. Symptoms were relieved and the endotracheal tube was removed within 4 h. Massive hemoptysis occurred in two patients during stent removal in the TF group. One patient died of asphyxia caused by massive hemorrhage and bleeding ceased in the other patient after administration of pituitrin. Two patients died perioperatively in the TS group, resulting in a clinical success rate of 94.7% (72/76). One patient with tracheal stenosis after resection of esophageal carcinoma died of massive hemoptysis 3 days after stent removal. The other patient died of respiratory failure 2 days after insertion of a second stent.

### Stent replacement after removal

Eight stents were replaced immediately and five stents were replaced 5 to 9 days after removal in the TF group. Replacements included Y-type tracheal stents (*n* = 5), L-type tracheal stents (*n* = 3), Y-shaped single-plugged stents (*n* = 2), straight tracheal stents (*n* = 2) and 1 combination of a large and a small Y-type tracheal stent. One patient required stenting due to proximal re-obstruction of the stent after removal of a straight tracheal stent, and the same size straight tracheal stent was implanted immediately. In TS group, 20 tracheal stents (15 straight tracheal stents and 5 Y type tracheal stents) were implanted again after removal for replacement of bare stents (*n* = 3), or due to restenosis (*n* = 9), migration (*n* = 3), inadequate expansion (*n* = 3), and intolerance (*n* = 2).

### Follow up

**Fig. 3:**
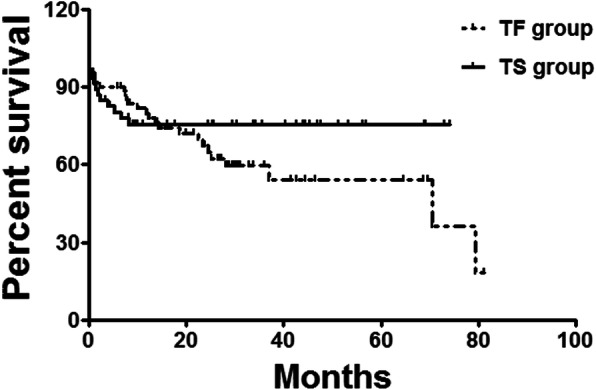
Survival rate follow up. The 0.5-, 3-, 6-year survival rates were 90.3, 59.6, and 36.1% for TF group, and 80.4, 75.7, 75.7% for TS group

All surviving patients were followed up. Five patients (6.0%) were lost during follow up in the TF group and four were lost (6.2%) in the TS group. Thirty-three patients were cured and 20 patients were improved in the TF group. Twenty-four patients died in the TF group, and tumor progression was the most common cause of death. One of the 24 patients died of respiratory failure due to asphyxia, and two patients died of massive hemoptysis and hematemesis. In the TS group, 29 patients were cured, 20 patients were improved and 6 patients underwent tracheotomy and tracheal T-tube implantation after stent removal (Table 4). The 0.5-, 3-, 6-year survival rates were 90.3, 59.6, and 36.1% for TF group, and 80.4, 75.7, 75.7% for TS group (Fig. 3).

**Table 4:** Clinical efficacy and death evaluation during follow-up

	TF group	TS group	*p*
Loss of follow up	5/84 (6.0%)	4/65 (6.2%)	1.0000
Clinical efficacy evaluation, N (%)			
Cured	33 (39.3%)	29 (44.6%)	0.6154
Improved	20 (23.8%)	20 (30.8%)	0.3576
Invalid	2 (2.4%)	3 (4.6%)	0.6534
Death	24 (28.6%)	9 (13.8%)	0.0458
Causes of death, N (%)			
Tumor progression	21 (25.0%)	5 (7.7%)	0.0081
Asphyxia	1 (1.2%)	4 (6.2%)	0.1679
Massive bleeding	2 (2.4%)	0 (0.0%)	0.5048

*N* number

## Discussion

Tracheal resection with primary anastomosis is the standard of care for the treatment of tracheal stenosis. However, tracheal resection is not suitable for long segmental stenosis, which also is associated with the complication of tracheal restenosis after resection. In this study, most of the patients enrolled had conditions which were complicated by underlying diseases and were not candidates for resection under general anesthesia. Tracheal stents are widely used clinically for the treatment of tracheal fistula [4–7] or tracheal stenosis [8–13]. However, tracheal stent placement may lead to a series of complications, and stent removal is sometimes necessary [23]. Currently, a tracheal stent is commonly removed under bronchoscopic guidance and general anesthesia [17, 24–27] or local anesthesia [28, 29]. Few studies have reported fluoroscopic removal of tracheal stents [12, 21, 22], and no study has compared the long-term outcomes and complications associated with fluoroscopy-guided removal of tracheal stents inserted for tracheal fistula or tracheal stenosis.

In the present study, 97.4% of stents implanted for tracheal stenosis were successfully removed under fluoroscopic guidance, which is higher than that reported by Verma et al. for silicon stent removal in patients with tracheobronchial stenosis [30]. Additionally, in their study 83.3% of stents were removed in one piece and only one stent showed retained stent pieces. In a study by Lunn et al., 73% of stents were removed piecemeal with rigid alligator forceps [24]. Our data indicate that fluoroscopy-guided removal of tracheal stents is a feasible procedure for tracheal fistula and tracheal stenosis.

Although the process of fluoroscopy-guided removal of tracheal stents is rapid, serious complications may occur during stent removal, such as massive bleeding, acute tracheal obstruction, and even death [24, 31, 32]. In our study, one patient patient in the TF the group died of asphyxia caused by massive hemorrhage. In the TS group, one patient died of massive hemoptysis 3 days after stent removal, and another patient died of respiratory failure. The overall perioperative mortality of stent removal under fluoroscopy was 1.97%, indicating that the technique was safe. In addition, the total incidence of complications was 21.1 and 22.4%, respectively, for the TF and TS groups. Compared with previous reports of stent removal by rigid bronchoscopy [24, 25, 29, 31], we showed a lower rate of complications.

Stent placement and removal with rigid bronchoscope under general anesthesia remains gold standard. Bronchoscopic technique has the advantage of direct visualization of tumor recurrence which can avoid some complications including hemoptysis in patients with malignancy. Theoretically, these two patients may be saved if bronchospcopic hemostasis was uesd. It should be noted that clinicians should pay attention to the risk of hemoptysis for patients with malignant tumors and, if necessary, a combination with endoscopic hemostasis should be used to further improve its safety. By contrast, fluoroscopic placement and removal of metallic stents may be more suitable for elder patients and patients who combined with severe co-morbidities or refuse general anesthesia.

Questions remain about the length of time that an indwelling stent should remain in place before removal in the treatment of tracheal disease [25]. The incidence of stent restenosis induced by granulation tissue hyperplasia and stent fracture may increase as indwelling time increases [33–35], and may lead to difficulty or failure of stent removal [28]. According to our findings, excessive granulation tissue was found 142.1 ± 25.9 days after stenting for tracheal fistula, and 89.9 ± 15.0 day after stenting for tracheal stenosis. Interestingly, tracheal stents were routinely removed about 3 months after implantation in our study. An indwelling interval of one to 3 months should be recommended for stent removal to avoid long-term complications.

There are weaknesses in our study. It was a retrospective study conducted in a single center. We solely performed stent removal under fluoroscopic guidance. In future studies, stents should be removed by a variety of technologies including bronchoscopy.

## Conclusions

Fluoroscopic removal of tracheal stent is safe and effective for both tracheal fistula and tracheal stenosis, with no significant difference. Recurrence of fistula or stenosis requiring stenting was the most comment complication. Clinicians should pay attention to the risk of hemoptysis for patients with malignant tumors and a combination with endoscopic hemostasis may help improve its safety.

## Supplementary Information

**Additional file 1:** **Video 1.** Stent removal for straight tracheal stent.

**Additional file 2:** **Video 2.** Stent removal for Y-type tracheal stent.

## Data Availability

The datasets used and/or analyzed during the current study are available from the corresponding author on reasonable request.

## Abbreviations

CT: Computed tomography
TF: Tracheal fistula
TS: Tracheal stenosis
